# Assessing the Impact of Different Technological Strategies on the Fate of *Salmonella* in Chicken Dry-Fermented Sausages by Means of Challenge Testing and Predictive Models

**DOI:** 10.3390/microorganisms11020432

**Published:** 2023-02-08

**Authors:** Anna Austrich-Comas, Anna Jofré, Pere Gou, Sara Bover-Cid

**Affiliations:** 1 Food Safety and Functionality Program, IRTA, Finca Camps i Armet, E-17121 Monells, Spain; 2 Food Quality and Technology Program, IRTA, Finca Camps i Armet, E-17121 Monells, Spain

**Keywords:** biopreservation, corrective storage, food safety, high-pressure processing, fermented meats

## Abstract

*Salmonella* is the main relevant pathogen in chicken dry-fermented sausages (DFS). The safety of shelf-stable DFS must rely on the production process, which should not only prevent growth but promote inactivation of *Salmonella*. The aim of the study was to assess the behaviour of *Salmonella* during the production process of two types of low-acid chicken DFS. The impact of the use of starter culture, corrective storage and high-pressure processing (HPP) at different processing times was assessed through challenge testing, i.e., inoculating a cocktail of *Salmonella* into the meat batter (at 6 Log_10_ cfu/g) used for sausage manufacture. Sausages of medium (*fuet*-type, FT) and small (snack-type, ST) calibre were elaborated through ripening (10–15 °C/16 d) and fermentation plus ripening (22 °C/3 d + 14 °C/7 d). Physico-chemical parameters were analysed and *Salmonella* was enumerated throughout the study. The observed results were compared with the simulations provided by predictive models available in the literature. In FT, a slight decrease in *Salmonella* was observed during the production process while in ST, a 0.9–1.4 Log_10_ increase occurred during the fermentation at 22 °C. Accordingly, DFS safety has to be based on the process temperature and water activity decrease, these factors can be used as inputs of predictive models based on the gamma-concept, as useful decision support tool for producers. *Salmonella* lethality was enhanced by combining HPP and corrective storage strategies, achieving >1 and 4 Log_10_ reductions for FT and ST, respectively.

## 1. Introduction

Chicken dry-fermented sausages (DFS) are innovative ready-to-eat (RTE) dry-cured meat products developed in recent years in response to consumer demands for foods with a reduced fat content. In this context, the use of skinless chicken meat instead of pork and/or beef generates a product with improved nutritional traits, at a lower cost than red meat and allows addressing some religious and cultural restrictions related to pork and beef [1]. The production process of DFS involves a series of hurdles (salting, curing agents, use of starter cultures, fermentation, drying), which sequentially select the desired competitive microbiota and inhibit pathogenic and spoilage bacteria. As finished products, DFS are considered shelf-stable meat products not requiring refrigeration [2]. However, bacterial pathogens can be present in raw meat and natural casings [3], survive the manufacturing process and remain present at unacceptable levels in the final product until the end of storage [4,5,6]. Accordingly, the food safety of DFS must rely on the production process, which should not only inhibit the growth of pathogenic bacteria but achieve the inactivation of relevant pathogens to a sufficient extent. In low-acid Mediterranean DFS, microbiological safety is primarily based on the low water activity (a_w_) of the final product [7]. Therefore, insufficient drying, e.g., associated with the shortening of the ripening period to increase profitability [7,8], can compromise the food safety of final products. Several notifications of withdrawal of DFS contaminated with *Salmonella* have been recorded in the last years in the EU Rapid Alert System for Food and Feed [RASFF alerts 2018.1111; 2020.3378; 2021.3787]. Additionally, several salmonellosis outbreaks involving DFS have been reported worldwide in the last decades, some of them involving low-acid DFS [9,10]. One of the main pathogens associated with raw chicken meat is *Salmonella* spp. [11,12], showing a high prevalence of up to 61% on chicken carcasses [13] and 18.1% on raw chicken meat [14].

Therefore, in the design and validation of the production process of chicken DFS, it is of the utmost importance to explore technological strategies for enhancing the inactivation of *Salmonella*. Different technological strategies might be applied to improve the food safety of DFS in relation to *Salmonella.* Starter cultures may provide a faster pH drop, which has been reported to improve product safety [12,15,16,17,18]. Corrective storage of the end-product before launching it to market is a low-cost strategy that takes advantage of the metabolic exhaustion of pathogens exposed to growth limiting a_w_ values at room temperature [5]. High-pressure processing (HPP) as a non-thermal technology to inactivate pathogens with minimal impact on the taste, flavour or nutrient content of the processed foods, have also been proposed for pork DFS [19]. Several studies have demonstrated that different factors affect the efficacy of HPP, such as low a_w_ and a high fat content [7,20,21]. However, given the lack of studies evaluating the performance of the aforementioned strategies in chicken DFS, product-specific studies (challenge tests) are necessary to assess their impact. Experimental studies can be complemented by predictive microbiology tools, which use mathematical models to assess the behaviour of pathogens under specific conditions using the physicochemical characteristics (such as pH, a_w_ or temperature) as inputs factors [22,23,24]. Stakeholders can use available models to assess the risks and, consequently, implement the strategies that are expected to be most effective in enhancing the food safety of DFS [25].

In this framework, the aim of the present study was to assess the fate of *Salmonella* during the production process of low-acid chicken DFS of medium (*fuet*-type, FT) and small (snack-type, ST) calibre, formulated without or with a lactic acid bacteria (LAB) starter culture. Besides the experimental challenge test approach, different predictive models available in the literature about *Salmonella* behaviour in DFS were applied. The simulations were compared with the experimentally observed results in order to identify a suitable tool that could be used for further assessments. In addition, the effect of strategies such as a corrective storage at 15 °C (compared with cold storage at 4 °C) and HPP applied at different steps of the processes were evaluated.

## 2. Materials and Methods

### 2.1. Bacterial Strain and Culture Preparation

A cocktail of three strains of *Salmonella enterica* from IRTA-Food Safety Program’s culture collection and isolated from pig or pig meat products were used to inoculate DFS: CTC1003 (serotype London) [5,26], GN0085 (serotype Typhimurium) [27] and CTC1754 (serotype Rissen) [5]. Each strain was independently grown in Brain Heart Infusion (BHI) broth (Beckton Dickinson, Sparks, NV, USA) for 24 h at 37 °C and 20% glycerol cryopreserved at −80 °C. 

*Latilactobacillus sakei* CTC494, a bioprotective starter culture from the IRTA’s collection was used as LAB starter culture [28]. It was grown anaerobically in Man-Rogosa-Sharpe (MRS) broth at 30 °C for 24 h and cryopreserved at −80 °C with 20% glycerol.

### 2.2. Chicken-Based DFS Preparation, Processing and Storage Conditions

Raw chicken meat (26 kg), already minced, was obtained from a DFS producer 12 h before the experiment and was kept at 0 °C until used. Raw chicken meat was inoculated with the cocktail of *Salmonella* strains (1% *v/w*) prepared by mixing equal amounts of each strain (thawed stock cultures at *ca*. 9 Log_10_ cfu/g; see Section 2.1) diluted in saline solution (0.85% NaCl and 0.1% Bacto Peptone) to achieve a concentration of *ca*. 6 Log_10_ cfu/g. The meat batter was homogenised for 75 s (mixing machine Mix-35P, Tecnotrip, Spain). The other ingredients, directly provided by the DFS producer, included glucose syrup, maltodextrin, NaCl, spices, sodium ascorbate, beet concentrate, flavour, nitrates and nitrites and a starter culture of *Staphylococcus xylosus* (Lyocarni SXH-38, Sacco System, Cadorago, Italy). They were also provided by the DFS producer and were added according to their recipe and mixed for an additional 135 s. In half of the batter *L. sakei* CTC494 was also added at *ca*. 6 Log_10_ cfu/g and mixed for 90 s. Sausages were stuffed (H15 stuffer, Tecnotrip, Terrassa, Spain) in natural pork casing of 40–42 mm diameter to produce *fuet* type (FT) or in edible collagen casing of 14 mm diameter to produce snack-type (ST). *Penicillum nalgiovensis* (Meat Surface PS 521, Lallemand Specialty Cultures, La Ferté-sous Jouarre, France) was applied on the surface of sausages by dipping into a spore solution.

**Figure 1:**
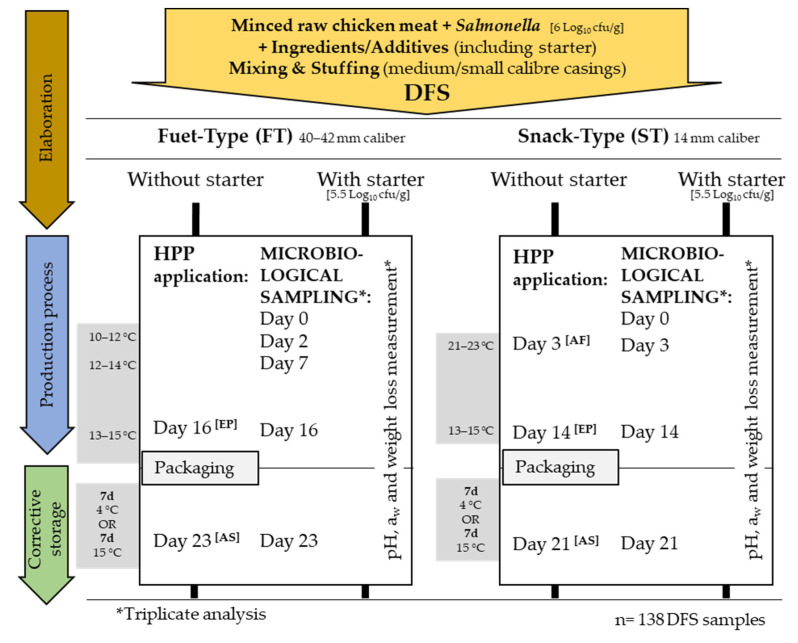
Manufacturing process and experimental design of *fuet*-type (FT) and snack-type (ST) dry-fermented sausages; *n:* total number of sampled sausages; *d:* days; DFS: Dry-Fermented Sausage; HPP: High-Pressure Processing; AF: After Fermentation; EP: End of Process; AS: After Storage.

According to the industrial conditions applied by the DFS producer, the production process (fermentation and ripening) was different for each type of DFS. FT was slightly fermented for a period of 2 days at 10–12 °C/76–80% Relative Humidity (RH) plus 5 days at 12–14 °C/81–86% RH, and a final ripening of 9 days at 13–15 °C/64–70% RH was applied. ST was fermented for 3 days at 21–23 °C/77–80% RH and subsequently ripened 11 days at 13–15 °C/64–70% RH. End products were put in PA/PE plastic bags (low water vapour permeability (2.8 g/m^2^/24 h) and oxygen permeability of 50 cm^3^/m^2^/24 h; Sistemvac, Estudi Graf, Girona, Spain), thermosealed with air inside the package and subsequently stored at 4 or 15 °C for up to 7 days (Figure 1).

### 2.3. High-Pressure Processing

Before HPP, DFS were put into the previously mentioned PA/PE bags and vacuum-packaged (EV-15-2-CD; Tecnotrip equipment, Terrassa, Spain). HPP consisted of 600 MPa for 5 min in a Wave6000 (Hyperbaric, Burgos, Spain) equipment at a starting temperature of 10 °C. The average pressure increase was 177 MPa/min and release was almost immediate. HPP was applied to the end product [EP] (day 14 for ST and day 16 for FT) and at the end of storage at 7 days at 4 and 15 °C [AS] (thermosealed with air). Additionally, in ST, HPP was also applied after the fermentation step [AF] (day 3), once the LAB had reached the maximum population density (stationary phase) (Figure 1).

### 2.4. Microbiological and Physicochemical Determinations

Microbiological analyses were performed in triplicate at the sampling points described in Figure 1: on day 0 (just after stuffing), during the production (before changing processing temperature on day 2, 7, 16 and 23 for FT and day 3, 14 and 21 for ST) and after storage. In total, 138 data points distributed all along the challenge test were obtained.

Product a_w_ was measured with an AquaLab^TM^ Series 3TE instrument (Decagon Devices Inc., Pullman, WA, USA). The pH was determined with a penetration probe (PH25 pHmeter) and 52–32 electrode (Crison Instrument SA, Alella, Spain). Lactic acid (D- and L- lactic acid, in g/100 g) was quantified with the D-/L-Lactic Acid (D-/L-Lactate) Assay kit (Megazyme International, Wicklow, Ireland) according to manufacturer instructions.

To enumerate LAB and *Salmonella*, 15 g of chopped product was ten-fold *w/v* diluted and homogenized in saline solution (0.85% NaCl and 0.1% Bacto Peptone) for 60 s in a Smasher^®^ (bioMérieux, Marcy-l’Étoile, France). This initial dilution was subsequently 10-fold serially diluted in saline solution. LAB counts were determined in Man-Rogosa-Sharpe (MRS) agar plates (Merck, Darmstadt, Germany) anaerobically incubated for 72 h at 30 °C in sealed jars with AnaeroGen sachet (Oxoid Ltd.) [29]. *Salmonella* was enumerated on chromogenic agar (CHROMagar^TM^ Salmonella Plus; Scharlab, Spain) incubated for 48 h at 37 °C [5]. Samples with expected *Salmonella* counts below the quantification limit (<10 cfu/g), were enriched in TSBYE at 37 °C for 48 h and the detection/non-detection of the pathogen was determined by plating on CHROMagar^TM^ Salmonella Plus.

### 2.5. Statistical Analysis of Analytical Results

The *t*-test (JMP 16, SAS Institute, Cary, NY) was used to test the differences of *Salmonella* and LAB counts, pH, a_w_ and lactic acid concentration between DFS types and to test the effect of the addition of starter culture, corrective storage or HPP. The significance level was established at *p* < 0.05.

### 2.6. Simulation of the Behaviour of Salmonella

Three predictive models published in the literature were used to simulate the behaviour of *Salmonella* during the production process of both types of DFS (i.e., FT and FT) as a function of the factors considered by each model. Hwang et al.’s [30] approach evaluates the non-thermal inactivation of *Salmonella* based on polynomial models for each process step on *soudjouk*-style fermented sausage. The input factor for the first step is the pH at the end of the fermentation while the input factors for the second step are the pH at the end of fermentation and the a_w_ at the end of ripening. Pin et al.’s [31] and Coroller et al.’s [32] models are based on the gamma-approach, which is able to simulate growth of *Salmonella* and, when the combination of given factors does not support growth, inactivation is simulated through Arrhenius-type [31] and Weibull [32] models. Both models use physicochemical parameters (pH and a_w_) and processing conditions (temperature) as input factors, and Coroller et al. [32] also consider the lactic acid concentration. All simulations were carried out using MS-Excel implementing the mathematical equations available in the publications.

## 3. Results

### 3.1. Physicochemical Characteristics of Fuet-Type (FT) and Snack-Type (ST) DFS

The physicochemical characteristics of FT and ST during the production process are shown in Table 1. In FT without starter culture, the pH decreased very slightly to 5.84, which is related to a relatively low increase in lactic acid concentration from 0.79 to 1.07 g/100 g. On the contrary, with the addition of a LAB starter culture (*L. sakei* CTC494), a higher acidification was observed at the end of the ripening (pH 5.11, lactic acid 3.49 g/100 g). The a_w_ of FT batches declined slowly to *ca*. 0.870 and the weight loss increased up to *ca*. 53% due to the drying process, without significant differences (*p* > 0.05) between batches of FT without and with starter culture.

The process of ST was 2 days shorter than that of FT and included a 3-day fermentation phase at higher temperature (21–23 °C), resulting in a fast pH drop to 5.32 and 5.05 in sausages without and with starter culture, respectively, at the end of the fermentation step (Table 1). In parallel, lactic acid concentration increased, reaching a maximum of *ca*. 2.1% for both batches at the end of fermentation (day 3) (Table 2). During the subsequent ripening period, the pH increased to remarkably high levels (>7) and the lactic acid concentration decreased. Both types of ST sausages showed higher a_w_ (0.926–0.915) and lower weight loss (*ca.* 49%) than FT at the end of the production process (*p* < 0.05). During the storage, pH, lactic acid concentration, a_w_ and weight loss did not change significantly (*p* > 0.05) for any batch.

### 3.2. Behaviour of LAB and Salmonella during the Production Process

In FT without starter culture, endogenous LAB progressively increased from 4 Log_10_ cfu/g in the meat batter to 7.4 Log_10_ cfu/g at the end of the production process (Table 2). In contrast, the addition of a *L. sakei* starter culture produced a rapid increase in LAB levels to >8 Log_10_ cfu/g in only two days despite the relatively low temperature applied for FT sausages. Due to the 22 °C fermentation step in ST sausages, a remarkably faster increase in LAB was observed in ST compared to FT. This difference was particularly noticeable in DFS without starter culture, where, two days after fermentation, endogenous LAB levels were 3.4 Log_10_ higher in ST than in FT. In the ST product, LAB levels were similar in batches elaborated without and with starter culture.

**Figure 2:**
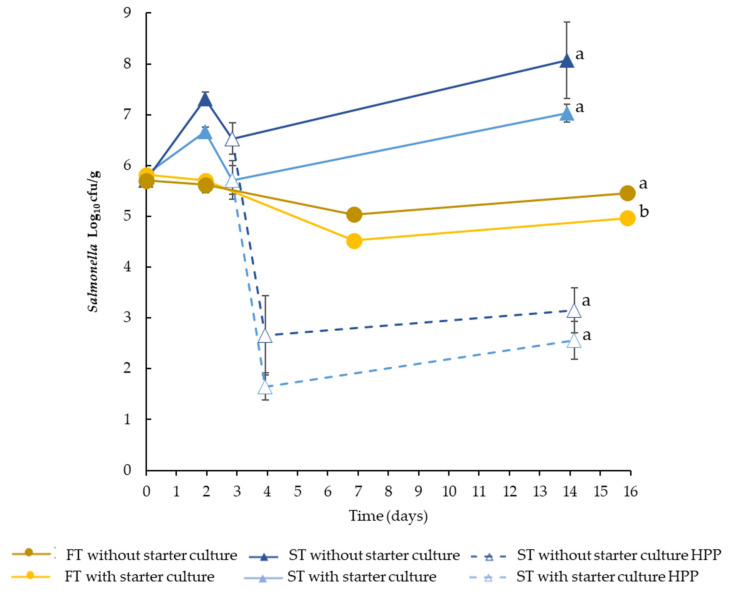
Counts (Log_10_ cfu/g) of *Salmonella* during the production process of *fuet*-type (FT) and snack-type (ST) without and with starter culture and/or HPP. Dashed lines represent HPP samples at day 3. Error bars: standard deviation of three replicates. For each type of product (without and with starter), values at the end of process with a different letter are significantly different (*p* < 0.05).

The behaviour of *Salmonella* during the production process depended on the type of DFS and the use of LAB starter culture (Figure 2). In FT, the pathogen showed a slight inactivation (*p* < 0.05) with a maximum Log_10_ reduction at the end of the production process of 0.25 and 0.75 in batches elaborated without and with starter culture, respectively. On the contrary, ST, fermented at 22 °C, allowed the growth of *Salmonella.* An increase in the levels of *Salmonella* were also observed during the ripening of ST sausages, particularly without starter culture, which can be related to the slightly higher pH and lower lactic acid concentration compared to ST with starter culture (Table 1).

### 3.3. Impact of Corrective Storage and HPP on Salmonella

**Figure 3:**
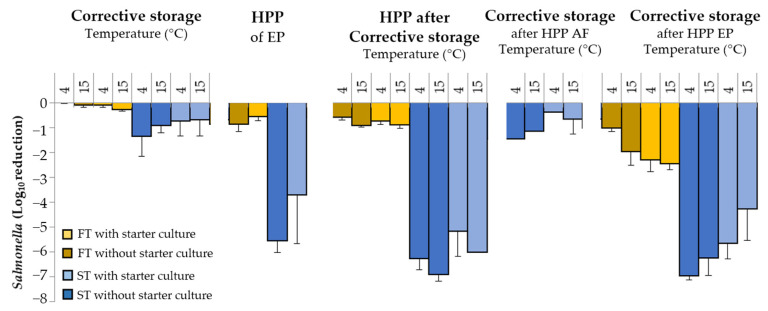
Inactivation of *Salmonella* (Log_10_ reduction) of *fuet*-type (FT) and snack type (ST) without and with the addition of starter culture due to corrective storage (7 days at 4 or 15 °C) and/or HPP. Time of application of HPP and sampling are indicated. AF: After Fermentation; EP: End of Process. Error bars correspond to standard deviation of three replicates. For each type of product, a significant effect of the applied strategy is indicated with an asterisk.

In FT sausages, storage temperature was not relevant (<0.3 Log_10_) for the reduction in *Salmonella* (Figure 3). In contrast, in ST sausages the pathogen reduction was enhanced by the corrective storage (0.69 to 1.35 Log_10_ reduction) without significant differences between starter culture application or storage temperature (*p* > 0.05).

HPP efficacy on DFS was evaluated at three different stages: after fermentation in ST (Figure 2 and Figure 3), at the end product (in FT and ST) and after the corrective storage (in FT and ST), and *Salmonella* was enumerated in the end product (before and after HPP) and after storage (Figure 3).

The immediate inactivation due to the application of HPP in the end product caused less than 1 Log_10_ reduction in FT products (*p* < 0.05). In ST, the HPP caused a much higher inactivation of *Salmonella,* achieving a 5.56 and 3.32 Log_10_ reduction in DFS without and with starter culture, respectively. Similar results were obtained when HPP was applied at the end of the corrective storage. Only a slight immediate inactivation (<1 Log_10_ reduction) was observed in FT sausages without differences between the storage temperature and the use of starter culture. Remarkable inactivation was obtained in ST without (6.28–6.91 Log_10_) and with (5.17–6.03 Log_10_) starter culture. 

For ST sausage, HPP application just after the fermentation caused a reduction in *Salmonella* of *ca*. 4 Log_10_ and after the corrective storage produced an additional inactivation of slightly more than 1 Log_10_ in both DSF without and with starter (Figure 2). A lower inactivation (≤0.6 log_10_) was observed in ST with starter culture stored at 4 and 15 °C.

Finally, in DFS submitted to HPP at the end of the process, the inactivation of *Salmonella* after storage (4 and 15 °C), was remarkable and higher than that observed immediately after HPP. In FT without starter culture, reductions of 1.02 and 1.97 Log_10_ were observed after storage at 4 and 15 °C, respectively (*p* < 0.05). When starter culture was added, a reduction in *ca*. 2.4 Log_10_ was observed, without differences between storage temperatures (*p* > 0.05). Conversely, in ST, the total inactivation of *Salmonella* was higher than in FT, with reductions of 6.3 and 7.0 Log_10_ after storage at 4 and 15 °C, respectively, without starter culture and 5.7 and 4.3 Log_10_ with starter culture.

### 3.4. Simulation of the Fate of Salmonella during the Production of DFS

**Figure 4:**
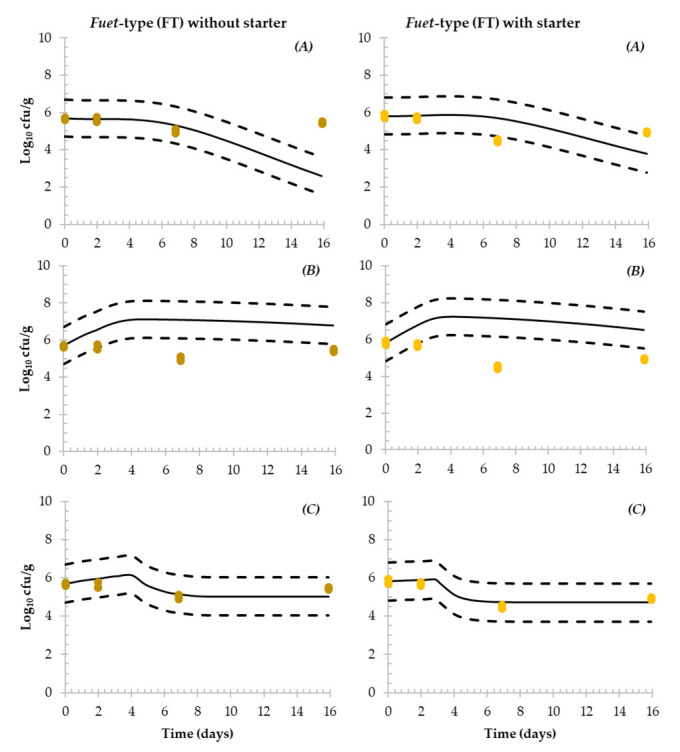
Simulation of *Salmonella* fate during the production process of *fuet*-type DFS without (**left**) and with (**right**) starter culture. Simulations were performed in MS-Excel implementing the (**A**) Hwang et al. [30]; (**B**) Pin et al. [31] and (**C**) Coroller et al. [32] predictive models (continuous line). Dashed lines represent the acceptable simulation zone (ASZ) ± 1 Log_10_ interval and dots represent the observed *Salmonella* counts obtained in the challenge test.

**Figure 5:**
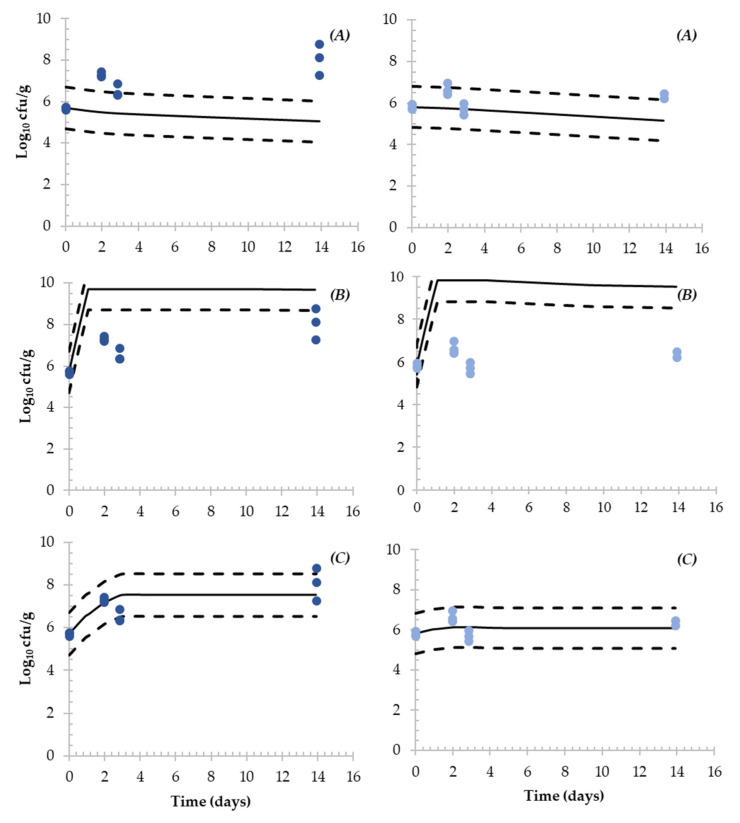
Simulation of *Salmonella* fate during the production process of snack-type DFS without (**left**) and with (**right**) starter culture. Simulations were performed in MS-Excel implementing the (**A**) Hwang et al. [30]; (**B**) Pin et al. [31] and (**C**) Coroller et al. [32] predictive models (continuous line). Dashed lines represent the acceptable simulation zone (ASZ) ± 1 Log_10_ interval and dots represent the observed *Salmonella* counts obtained in the challenge test.

The behaviour of *Salmonella* observed during the challenge was compared with three different predictive models available in the literature specifically developed for *Salmonella* in dry-fermented sausages (Figure 4 and Figure 5).

Considering Hwang et al.’s [30] model, a greater inactivation of *Salmonella* was simulated for FT compared with the experimentally observed results, producing fail-dangerous predictions (overestimation of the inactivation) (Figure 4). Regarding ST (Figure 5), the underestimation was more evident as experimental results showed growth and a slight inactivation (*ca.* 0.65 Log_10_ reduction).

The model proposed by Pin et al. [31] predicted a considerable growth during the first days of the production process in FT and particularly in ST sausages. In FT without and with starter culture, an increase of *ca*. 1 Log_10_ in 14 days was simulated. Subsequently, a slight inactivation was simulated for both batches (*ca.* 0.6 Log_10_ reduction in 14 days). Regarding ST, 3.7 and 4 Log_10_ increase were simulated for ST without and with starter culture, respectively, during the first day of fermentation, followed by a slight inactivation. The simulated behaviour resulted in greater *Salmonella* levels than the observed values during the challenge test (overestimation, fail-safe).

Finally, Coroller et al.’s [32] predictions in FT showed 0.33 and 0.20 Log_10_ increases followed by a slight inactivation of *Salmonella.* For both batches, good agreement between the model and the observed values was obtained (inside the ASZ of ±1 Log_10_). Regarding ST, the simulation was also in agreement with the observed results. In spontaneously fermented product and when a starter culture was added, 1.84 and 0.32 Log_10_ increases, respectively, were simulated followed by neither growth nor inactivation, as the observed values inside the ASZ of ±1 Log_10_.

**Figure 6:**
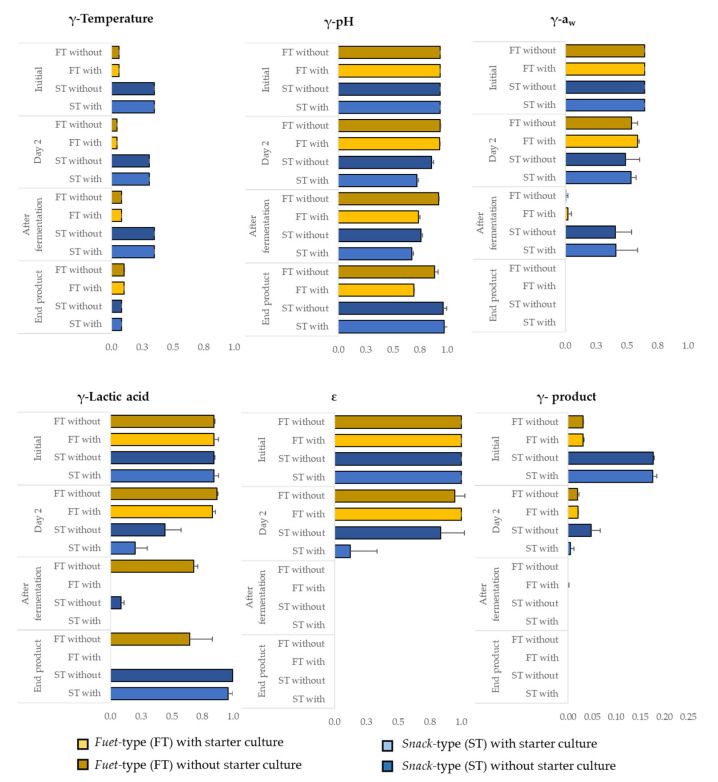
Gamma (γ) values for each environmental factor, the interaction term (ε) and the overall gamma product (γ product) considered in the model proposed by Coroller et al. [32] to simulate *Salmonella* behaviour in DFS: initial time (day 0), at day 2, after fermentation (day 3 for ST and day 7 for FT) and end product (day 14 for ST and day 16 for FT). Error bars correspond to the standard deviation of the values.

The contribution of each individual factor (pH, a_w_, temperature and lactic acid) and the interaction term (ε) resulting from the model proposed by Coroller et al. [32] on the inhibition of *Salmonella* growth was quantified through the individual gamma (γ) estimates and the overall gamma product (Figure 6). The lower the γ value, the stronger the inhibitory effect (i.e., for γ = 0, growth is totally inhibited, while γ = 1 indicates no inhibitory effect at all). At the initial stage (day 0), temperature is the main limiting factor for *Salmonella* growth for FT and ST, both without and with starter culture. Regarding ST, the greater γ product obtained was due to the highly inhibitory effect of the γ temperature at day 0. While the γ pH was relatively high in all types of products (less contributing factor of the *Salmonella* growth inhibition), the accumulation of lactic acid after the fermentation had a clearly higher impact in sausages with starter culture. This biopreservation effect of the starter culture was the key inhibitory factor for ST sausages. The contribution of the lowered a_w_ becomes relevant until the end product for all batches, making the ε fall to 0 (preventing growth).

## 4. Discussion

### 4.1. Effect of Formulation and Production Process on Salmonella

The safety of DFS should rely on the control of the production process. In low-acid DFS produced in the Mediterranean countries, usually produced at moderately low temperatures (e.g., 15 °C) and often without acidifying (i.e., no LAB) starter culture [33], the pH is not always a sufficient hurdle and the process temperature and drying (a_w_) are the main factors contributing to their food safety [7]. In these types of products, the application of a LAB starter culture able to grow at low temperatures is one of the strategies that can be applied at production level to reduce *Salmonella*.

Regarding LAB growth, the differences between FT and ST chicken DFS formulated without and with starter culture can be explained by the production process conditions (i.e., temperature and relative humidity), which generated different acidification profiles. In ST, the greater growth of LAB (>8 Log_10_ cfu/g at day 2 in products with and without starter) could be associated with a higher temperature (21–23 °C) during the first days of the production process compared with FT, which were fermented at a temperature of <15 °C and showed a delayed LAB growth (5 Log_10_ cfu/g), and neither pH decreases nor lactic acid production at day 2 were observed. As fermentation temperature approached the optimal growth temperature of LAB (30 °C for *L. sakei,* [34]), pH decreased and lactic acid increased. Coroller et al. [32] used starter cultures with different acidifying capacity (0.5 and 1 unit reduction after 2 days of fermentation) and showed faster production of lactic acid (1 g/100 g) when the faster starter culture was used.

Due to the acidifying effect of the added starter culture, at the end of the production process DFS formulated with *L. sakei* showed lower pH and higher lactic acid concentrations (e.g., pH = 5.1 and 3.5% lactic acid in FT). The type of sausage also had a great impact on these physicochemical parameters. In ST, the production process conditions favoured the development of a thick layer of surface mould, which prevented their proper drying and generated a final product with a_w_ values greater than expected. Moreover, the pH of the final product was raised, which is associated with the breakdown of lactate and release of ammonia from proteins by the moulds, which together with their proteolytic and lipolytic activities play a role in the sensory properties of the moulded Mediterranean DFS [35,36].

Acidification is a relevant factor limiting the growth and enhancing the inactivation of *Salmonella* [6]. In accordance, results of the present study showed limited inactivation of *Salmonella* during the production process of low-acid products (0.9 Log_10_ in DFS of pH 5.11) and, remarkably, the ability of *Salmonella* to grow in non-acidified products fermented at 22–23 °C.

No publications have been found regarding the behaviour of *Salmonella* in chicken-based DFS, but numerous studies are available for other types of meat, especially pork, elaborated without and with LAB starter culture [5,32,37]. In accordance with present results, previous studies reported a limited reduction in *Salmonella* in low-acid DFS with relatively short ripening periods and possibility of growth at the initial fermentation phase. In this regard, Coroller et al. [32] also reported *Salmonella* growth up to 2 Log_10_ during the first 2 days of manufacturing at 23 °C and 87% RH in DFS, and according to Werlang et al. [38], *Salmonella* grew 1.61 Log_10_ in 5.8 days in Brazilian DFS with starter culture (mixture of *Staphylococcus* and *L. sakei*) fermented at 30 °C and 95–99% RH (pH = 5.4 at day 2.75).

Throughout the production process, Bonilauri et al. [39] reported 0.05 to 1.36 Log_10_ reductions of *Salmonella* depending on the pH value (pH < 5.4) at the end of the acidification step of 20 Italian salami types with different ripening profiles, showing that greater pH drops produced greater *Salmonella* reductions. Garriga et al. [37] reported a *ca.* 1.75 Log_10_ reduction in *fuet*-type DFS without and with starter culture associated with the combination of several hurdles (acidic conditions, curing agents and low a_w_ values). The impact of acidification through the addition of the chemical acidulant Glucono-delta-Lactone (GdL), which immediately decreases the pH in the meat batter, has also been evaluated, showing enhanced *Salmonella* reduction due to GdL application and especially when combined with a starter culture [40]. However, the levels of acidification achieved (down to pH = 4.7) are outside the range of low-acid DFS.

Considering that hurdles in food are somewhat interchangeable [41], in low-acid DFS a_w_ reduction becomes more important than acidification for controlling *Salmonella,* although the pathogen has been reported not to grow at a_w_ below 0.94 when other conditions are optimal [42,43]. The progressive drying (usually to levels below 0.92 in Mediterranean DFS) does not only aim to inhibit the growth but rather enhance the inactivation of *Salmonella* during the production process.

Higher inactivation of *Salmonella* in FT could also be partially attributed to the calibre size. Medium calibre DFS (e.g., FT) need more time to remove water and create adverse conditions for *Salmonella* compared to smaller calibre DFS (e.g., ST). This finding agrees with previously published results [31,39,44] reporting that *Salmonella* reduction or prevalence was associated with the length of the drying and the a_w_ of the end product, which were correlated with the calibre size.

### 4.2. Impact of Corrective Storage and HPP on Salmonella

The application of a corrective storage strategy in FT DFS did not provide additional inactivation of *Salmonella*. Similarly, Hwang et al. [30] reported no significant reduction in *Salmonella* when DFS (a_w_ between 0.92 and 0.86) were stored at 4, 21 and 30 °C up to 60 days. Nevertheless, other authors found that room temperatures (>15 °C) enhanced the inactivation extent of food-borne pathogens [5,45,46,47]. In this context, the limited effect of the corrective storage alone in FT (7 days at 4 and 15 °C) could be related to the low temperatures and the relatively short storage period. In DFS of higher a_w_, greater inactivation was observed. These results agree with Serra-Castelló et al.’s [5] findings, reporting higher reductions of *Salmonella* in *fuet* DFS (made of pork) with higher a_w_ (4 and 3.7 Log_10_ reduction after 60 days in DFS with a_w_ 0.93 and 0.90, respectively). Santillana-Farakos et al. [48] showed an increased survival capacity of *Salmonella* with decreasing a_w_ of the matrix in low-moisture foods. Thus, *Salmonella* could have acquired higher resistance due to the harsh conditions that progressively appear during the production process of DFS, which are more stressful in sausages with lower a_w_ at the end of the drying.

It is known that the efficacy of HPP in inactivating pathogenic bacteria depends on the physicochemical characteristics of the food matrix as well as the physiological status of microbial cells. In general, the greater efficacy of HPP in ST compared to FT at the end of the production process and at the end of the corrective storage could be partially attributed to the higher a_w_ of ST (0.926–0.915) than FT (<0.876) sausages, which exerted piezoprotection on *Salmonella* in FT and reduced the HPP inactivation [20,49,50]. This would be due to the stabilization of proteins (particularly enzymes) and the reduction of pressure-sensitive denaturation [20,51]. On the other hand, the pressure resistance of microorganisms is affected by their physiological status. In this context, greater pressure resistance has been observed for bacteria in the stationary phase than in the exponential growth phase [52,53,54]. Indeed, when cells grow at higher temperatures during the production process, they have less pressure resistance than at lower temperatures, due to a change in the fatty acid from membrane cells [52,53]. All of this could have contributed to make ST sausages (exponential phase and fermented at 22 °C) more susceptible to HPP than FT sausages (stationary phase and ripened <15 °C). Acidity has also been widely described to enhance high-pressure inactivation and compromise recovery [55], but it did not play a role in the evaluated low-acid DFS (pH >5).

### 4.3. Evaluation of Predictive Models Simulating the Fate of Salmonella in DFS

The model proposed by Hwang et al. [30] considers the pH at the end of fermentation and a_w_ at the end of ripening, but not the temperature of the process, which is known to be a key factor affecting pathogen behaviour [5]. This polynomial model was developed in a *soudjouk*-type DFS made of beef and with a pH after fermentation (4.6–5.2) lower than the one observed in this study (pH > 5). Moreover, the model can only simulate inactivation, so it was not possible to predict the growth observed in ST, generating fail-dangerous simulations.

The gamma-concept model followed by Pin et al. [31] considers pH, a_w_ and temperature and can simulate both growth and inactivation depending on the contribution of each input factor. However, this model overpredicted the growth of *Salmonella* at the early stages of the DFS elaboration process, simulating higher growth than that observed in the different batches of FT and ST. The model does not take into consideration the potential impact of lactic acid, known to considerably contribute to the growth inhibition of enteric pathogens.

Alternatively, the model developed by Coroller et al. [32], also based on the gamma-concept, considers lactic acid concentration as an additional input factor. The predictions of this model were much more in agreement with the observed experimental results compared with the previous models, probably because it was specifically developed to characterise the behaviour of *Salmonella* during the production process of DFS comparable to the ones evaluated in this study. Werlang et al. [38] also applied the Coroller et al. [32] model and successfully predicted the slight (*ca.* 1 Log_10_) growth of *Salmonella* during the first two days of salami fermentation (at 30 °C) and a subsequent inactivation (−4.9 Log_10_) during 36 days of ripening at 20 °C. The study also reported growth (1.16 Log_10_) during the first 2 days followed by inactivation (−4.87 Log_10_).

The quantification of the contribution of each gamma factor to the inhibition of *Salmonella* may be used to identify the combination of factors that prevent growth and promote inactivation. Given the importance of temperature and drying, producers of low-acid DFS should take these two relevant parameters into account when applying the safety-by-design concept in their products. The model can be used as a decision support tool, as good agreement was observed for all types of chicken DFS studied.

## 5. Conclusions

The safety of low-acid chicken DFS has to be assessed through the lethality of the production process. *Salmonella* behaviour during fermentation and ripening is influenced by the process conditions, which can promote the growth of the pathogen if fermentation is carried out at moderately high temperatures or enhanced inactivation during the progressively harsher environment during drying. In the evaluated low-acid DFS, the addition of a starter culture (*L. sakei*) turned out to be a low-impact strategy against *Salmonella* during the production process, the safety of which has to be based on the process temperature and the water activity decrease. In this context, a suitable predictive model based on the gamma-concept can be used as a decision support tool for DFS producers to identify and design the combination of elaboration conditions that inhibit the growth and promote the inactivation of *Salmonella*. Moreover, the process can be complemented with post-processing strategies such as the combined application of HPP and corrective storage.

## Figures and Tables

**Table 1:** Results of physicochemical determinations during the production process of *fuet* and snack-type DFS. Results are expressed as mean ± standard deviation of three replicates.

	Time (Days)	*Fuet*-Type		Snack-Type	
		without Starter	with Starter	without Starter	with Starter
pH	0	6.11 ± 0.00 ^a^	6.11 ± 0.00 ^a^	6.11 ± 0.00 ^a^	6.11 ± 0.00 ^a^
	2	6.14 ± 0.02 ^a^	6.09 ± 0.04 ^a^	5.69 ± 0.09 ^a^	5.20 ± 0.04 ^b^
	3	-	-	5.32 ± 0.05 ^a^	5.05 ± 0.04 ^b^
	7	6.04 ± 0.03 ^a^	5.24 ± 0.04 ^b^	-	-
	14	-	-	7.63 ± 0.33 ^a^	7.29 ± 0.02 ^a^
	16	5.84 ± 0.16 ^a^	5.11 ± 0.01 ^b^	-	-
a_w_	0	0.979 ± 0.000 ^a^	0.979 ± 0.000 ^a^	0.979 ± 0.000 ^a^	0.979 ± 0.000 ^a^
	2	0.974 ± 0.000 ^a^	0.977 ± 0.001 ^a^	0.972 ± 0.005 ^a^	0.974 ± 0.000 ^a^
	3	-	-	0.969 ± 0.006 ^a^	0.969 ± 0.008 ^a^
	7	0.945 ± 0.007 ^a^	0.944 ± 0.009 ^a^	-	-
	14	-	-	0.926 ± 0.009 ^a^	0.915 ± 0.013 ^a^
	16	0.876 ± 0.001 ^a^	0.864 ± 0.012 ^a^	-	-
Lactic acid (g/100 g)	0	0.79 ± 0.02 ^a^	0.81 ± 0.10 ^a^	0.79 ± 0.02 ^a^	0.81 ± 0.01 ^a^
	2	0.71 ± 0.01 ^a^	0.83 ± 0.04 ^a^	1.26 ± 0.18 ^a^	1.82 ± 0.04 ^b^
	3	-	-	2.13 ± 0.02 ^a^	2.19 ± 0.09 ^a^
	7	1.47 ± 0.09 ^a^	2.67 ± 0.21 ^b^	-	-
	14	-	-	0.54 ± 0.24 ^a^	1.38 ± 0.62 ^a^
	16	1.07 ± 0.34 ^a^	3.49 ± 0.31 ^b^	-	-
Weight loss (%)	0	0.00 ± 0.00 ^a^	0.00 ± 0.00 ^a^	0.00 ± 0.00 ^a^	0.00 ± 0.00 ^a^
	2	11.83 ± 2.02 ^a^	12.79 ± 2.73 ^a^	10.19 ± 6.91 ^a^	12.79 ± 5.66 ^a^
	3	-	-	18.09 ± 6.47 ^a^	20.87 ± 6.74 ^a^
	7	35.38 ± 4.20 ^a^	38.60 ± 1.91 ^a^	-	-
	14	-	-	49.41 ± 1.07 ^a^	48.81 ± 0.99 ^a^
	16	53.26 ± 0.73 ^a^	53.99 ± 0.27 ^a^	-	-

Values with different small letter in the same line and within the same DFS type (comparing DFS without and with starter culture) are significantly different (*p* < 0.05).

**Table 2:** Counts (Log_10_ cfu/g) of lactic acid bacteria during the production and after storage of *fuet*-type (FT) and snack-type (ST) DFS. High-Pressure Processing (HPP) was applied at day 16 and 23 in FT; and at day 3, 14 and 21 in ST. Std: Standard production process. Results are expressed as mean ± standard deviation of three replicates.

Time (Day)	*Fuet*-Type				Time	Snack-Type			
	without Starter		with Starter		(Day)	without Starter		with Starter	
	Std	HPP	Std	HPP		Std	HPP	Std	HPP
0	4.11 ± 0.03	-	5.67 ± 0.17	-	0	4.11 ± 0.03	-	5.67 ± 0.17	-
2	5.00 ± 0.05	-	8.41 ± 0.10	-	2	8.42 ± 0.43	-	8.68 ± 0.06	-
7	5.22 ± 0.38	-	8.17 ± 0.10	-	3	8.18 ± 0.22 ^a^	3.46 ± 0.47 ^b^	8.42 ± 0.08 ^a^	3.97 ± 1.16 ^b^
16	7.38 ± 0.46 ^a^	7.26 ± 0.19 ^a^	9.54 ± 0.08 ^a^	8.51 ± 0.07 ^b^	14	8.24 ± 0.44 ^a^	5.83 ± 0.40 ^b^	8.61 ± 0.05 ^a^	7.29 ± 0.43 ^b^
23	7.39 ± 0.24 ^a^	7.55 ± 0.29 ^a^	8.53 ± 0.11 ^a^	8.57 ± 0.07 ^a^	21	8.19 ± 0.38 ^a^	5.94 ± 0.21 ^b^	8.53 ± 0.09 ^a^	5.03 ± 0.95 ^b^

Values with a different letter for Std and HPP in the same line and within the same DFS type (*Fuet* or Snack) and starter treatment (without or with starter) are significantly different (*p* < 0.05).

## Data Availability

Data available on request.
